# Want-to, have-to, amotivation, grit, self-control, and tolerance ambiguity among university students: latent profile analysis

**DOI:** 10.1186/s40359-023-01298-w

**Published:** 2023-09-02

**Authors:** Amal Alhadabi, Ibrahim Al-Harthy, Said Aldhafri, Hussain Alkharusi

**Affiliations:** 1 Evaluation and Measurement, Psychology Department, College of Education, Sultan Qaboos University, Al-Khodh, Muscat, Sultanate of Oman; 2 Educational Psychology, DQAE Directorate, National University of Science and Technology, Bousher, Muscat, Sultanate of Oman; 3 Educational Psychology, Psychology Department, College of Education, Sultan Qaboos University, Al-Khodh, Muscat, Sultanate of Oman; 4 Evaluation and Measurement, Psychology Department, College of Education, Sultan Qaboos University, Sultanate of Oman, Al-Khodh, Muscat, Sultanate of Oman

**Keywords:** Academic motivation, Grit, Self-control, Demographics, University students, Latent profile analysis

## Abstract

The current study adopted a person-centered approach to identify distinctive university students’ profiles based on three variables (i.e., three academic motivations, grit, and self-control), regress multiple covariates (i.e., gender, age, study level, and college) on profile membership, and estimate differences on ambiguity tolerance across the estimated profiles. Data on 525 university students were modeled using Latent Profile Analysis. The findings found three latent profiles, which were: [1] *Unmotivated and undisciplined students with low grit*, [2] *Moderately motivated and disciplined students with average grit*, and [3] *Highly motivated, gritty and disciplined students*. Gender, study level, and college significantly predicted profile affiliation, identifying the characteristics of students within each profile. Significant differences were revealed in the ambiguity tolerance among the obtained profiles. These valuable results offer customized recommendations and prospective initiatives, strengthening the constructive effect of proper academic motivation types, purposeful grit, and intentional self-control (143 words).

## Introduction

While an active line of research has investigated the association among three academic motivations (i.e., want-to, have-to, and amotivation), grit, and self-control among university students [4, 5], the deep investigation of latent heterogeneity in the academic motivations, grit, and self-control among higher education students is quite underinvestigated [6, 7]. For instance, some gritty students with high self-control hold pure joy when learning new materials; others seek to be recognized as top achievers and showed the needed discipline and grit to accomplish any academic or personal task, while others show zero motives, grit, and self-control to accomplish specific tasks. In the same vein, students differ in dealing with setbacks, tasks that require long-term interest, and impulses that seem gratifying, but incongruent with academic goals (e.g., watching funny videos on TikTok, short clips about friends on Snapchat, or new celebrities’ photos in Instagram). Such divergence has prominent effects on students’ productivity and success in the academic and personal contexts (e.g., [2, 8, 9]).

An extensive literature review has displayed four main points. First, evident heterogeneity exists among university students in the three academic motivations, grit, and self-control (e.g., [6, 7, 10–12]). However, none of the prior studies has modeled this unobserved heterogeneity, particularly in the five attributes simultaneously, to identify distinct profiles (i.e., groups) of students that might provide a sharper understanding of the characteristics of each group and how it associates with students’ ability to tolerate ambiguity, particularly in an era of a huge uncertainty associated with Covid-19 [13]. For example, Hong et al. [6] showed four latent profiles of undergraduates based on four outcomes of academic motivation (i.e., self-efficacy, achievement goals, value, and cost). These profiles were [1] High cost (i.e., was characterized by high cost and low self-efficacy, goals, and values), [2]. Moderately Motivated (i.e., had students with an average level of all variables), [3] High goals (i.e., contained students with higher goals compared to other profiles), and [8]. Mastery-Driven (i.e., had students with low performance and avoidance goals).

Second, a large number of studies have followed a variable-centered approach when investigating academic motivation, grit, and self-control (e.g., [4, 14]). This line of research implies investigating the relationships among multiple variables (e.g., achievement, study strategies, procrastination, etc.) and variables of interest in the current study, as in most conventional analyses (e.g., regression and structural equation modeling). Much limited body of studies implemented a person-centered framework in exploring the existence of various profiles/subpopulations of students based on at least one variable of interest in this study (e.g., [6, 15–17]), and none of these prior studies has modeled latent heterogeneity on the combination of the five outcomes (i.e., three academic motivations, grit, and self-control) among university students. The central units of inquiry in the person-centered approach are the individuals. This framework identifies clusters of people by examining the associations between persons [18]. Thus, adopting the person-centered approach in investigating heterogeneity in five outcomes is a priority to acquire a clearer understanding and offer well-tailored suggestions.

Third, demographics (e.g., gender and age), besides other covariates, can explain this latent heterogeneity [6, 11, 19]. Nonetheless, the literature has provided a mixed bag of findings related to the role of demographic information on academic motivations, grit, and self-control profiles. Related to gender, Hong et al. [6] found that more males were classified in the high-cost and mastery-driven profiles than the moderately motivated and high-goals groups, implying that males were more likely to report lower performance and high perception of costs. In contrast, Stolk et al. [19] revealed that females reported less self-determined motivation compared with males. Unlike Hong et al. [6] and Stolk et al. [19], Litalien et al. [11] found no differences between profiles across gender. Concerning age, Hong et al. [6] found that older students exhibited a significantly higher likelihood of membership in the high goals and mastery-driven profiles compared to other profiles. Conversely, Litalien et al. [11] showed no differences between profiles across age groups. These findings imply that demographics can elucidate the characteristics of students within each profile.

Fourth, membership in the academic motivation profiles is associated with several distal outcomes. The distal outcomes vary across prior studies. Wang et al., [20] found that profile membership significantly predicted four distal outcomes (i.e., effort, value, competence, and time spent on Math beyond homework). Also, Vansteenkiste et al., [12] showed that profile membership predicted four learning outcomes (i.e., cognitive processing, and meta-cognitive regulation including test anxiety, time use, and meta-cognitive strategy), two features of determination (i.e., effort regulation and procrastination), and three dimensions of teacher need support (i.e., autonomy support, structure, and involvement). None of the prior person-centered studies examined the association between profile membership and ambiguity tolerance as a distal outcome. Nonetheless, several prior studies, which adopted the variable-centered approach, found that ambiguity tolerance is significantly associated with higher academic motivation [21] in online learning, positive emotion including determination and grit [22], intellectual curiosity and assertiveness [23] among university students.

Considering the above, the findings underline considerable latent variability in academic motivations, grit, and self-control, which can be ascribed to several demographic information. Simultaneously, modeling this variability can facilitate predicting other students’ outcomes, particularly their ability to tolerate ambiguity. Therefore, in an attempt to address the gaps mentioned above, the main interest of the current study was identifying to what degree the students hold different levels of academic motivations, grit, and self-control by modeling the latent heterogeneity, resulting in classifying students in various profiles. Furthermore, to what extent the personal demographic information accountable for the characteristics of the estimated profiles? In sequence, do the estimated profiles predict ambiguity tolerance?

## Literature review

### Academic Motivation

One of the essential determinants of goal attainment is what motivates students. The literature identifies three main types of academic motivation: intrinsic motivation (i.e., want-to), extrinsic motivation (i.e., have-to), and amotivation. These three types are rooted in self-determination theory [24]. Want-to-motivation, also known as intrinsic motivation, refers to the extent to which a person pursues a goal because it is enjoyable, meaningful, and connected to the person’s values. In contrast, have-to-motivation (i.e., controlled motivation) reflects the extent to which the goals are attained due to extrinsic incentives (e.g., social pressure and rewards) and internal introjected pressures (e.g., avoidance of guilt and shame; 70). The amotivation represents the state when the two former types are low and when an individual feels a lack of interest and questions the value of doing any task [25].

The intrinsic and extrinsic motivations are multidimensional constructs themselves [25]. Intrinsic motivation has three sub-dimensions, which include motives to know, motives toward accomplishment, and motives to experience stimulation (i.e., sensory pleasure about doing a certain task). Comparatively, the extrinsic motivations have three sub-factors, including identified regulation (i.e., knowing and feeling the benefit of doing a task), external regulation (i.e., doing a task due to external factors like social pressures and rewards), and introjected regulation (i.e., doing a task to maintain self-worth and avoid guilt and shame).

A burgeoning recent number of studies have shown want-to-motivation positively associated with higher achievement [26], better well-being [27], and pro-social behavior [28]. On the other hand, have-to-motivation is associated with less goal attainment [4], subjective perception of more disruptive obstacles [14], active procrastination [29], and greater response to temptation [9].

### Grit

Grit represents an assiduous pursuit of long-term targets regardless of hardships [30]. Meaning, gritty students show more diligence in surmounting barriers and lasting interest to fulfill their goals despite setbacks and lack of assistance. More recent empirical research has highlighted that grit is positively associated with numerous academic outcomes, including academic performance through numerous mediators (e.g., self-efficacy and achievement orientation goals; 3; 18), Mastery and performance-approach goals [2], intellectual engagement [31], metacognition [32], life satisfaction [33], and graduation rate [34]. A recent meta-analysis study revealed that grit correlated with undergraduate GPA, retention, intent to persist in college, college satisfaction, and self-efficacy [35].

### Self-Control

Milyavskaya and Inzlicht [36] define self-control as “the effortful inhibition of an immediately gratifying behavior or impulse” (p.11). Meaning, a person exerts effort when deciding to enjoy an immediate hedonic behavior (e.g., watching TV) versus completing less pleasurable tasks (e.g., studying) that facilitate long-term goals (e.g., completing an academic degree). Self-control requires a conscious, deliberate, and effortful act, which according to Werner and Milyavskaya [4] not necessarily result in achieving long-term goals. Rather it associates with pessimistic outcomes like ego depletion [37]. An influential line of research, though, has emphasized the positive role of self-control. For example, Tangney and colleagues [38] revealed that self-control positively correlated with better academic achievement, higher self-esteem, healthier diet, optimal emotional response, and quality relationships.

Self-control has essential influences on students’ academic outcomes. Duckworth et al., [39] emphasized that self-control has positive effects on academic achievement, academic attainment, course grades, standardized tests, accomplishing academic goal-congruent tasks, and dampening academic goal-incongruent tasks. A meta-analysis of 104 studies revealed significant positive associations between self-control and a set of desired behaviors surrounding school, work, eating habits, weight, interpersonal functioning, and well-being with small to medium effect sizes [40]. In addition, this study found a significant association between low self-control and a set of undesired behaviors, including deviant behavior (e.g., nonviolent crime, cheating, driving above the speed limit), unhealthy lifestyle (e.g., eating disorder symptoms and unsafe sexual behavior), and addictive behaviors (e.g., smoking and marijuana use), reflecting medium effect sizes.

### Ambiguity Tolerance (AT)

Budner [41] articulates tolerance to ambiguity as a tendency to perceive ambiguous situations as desirable and not a source of threat. For instance, students who tolerate ambiguity are more likely to engage effectively in challenging learning experiences, are more open to exploring new and complicated learning tasks, and practice more creative and critical thinking in such ambiguous learning experiences. Several studies have indicated that AT is associated with novelty [42], higher academic motivation [21] in online learning, positive feeling and life satisfaction [22], desirable personality type [43], students’ engagement [44], and intellectual curiosity and assertiveness [23]. Another study revealed that students with a moderate level of AT had higher reading comprehension scores compared to students with high and low AT, suggesting the presence of a relationship between AT and learning strategies [45]. Conversely, lack of AT is associated with higher levels of anxiety when receiving unstructured learning material [22, 46], less motivation to participate in online learning [21], and higher obsessive-compulsive response [47].

### Latent Heterogeneity in the Study Variables

As has been noted, the literature has pointed out the presence of considerable latent heterogeneity in the three academic motivations (want-to, have-to, and amotivation), grit, and self-control among university students. Not all students are eager to learn and do not approach academic tasks with the same level of discipline [39], implying that treating students in an even manner when using the variable-centered approach induces invalid findings and inaccurate inferences [11]. Using the person-centered framework would result in a further accurate diagnosis of the level of the current study’s variables in the estimated profiles, facilitating accurate recognition of students’ characteristics in each profile. However, no prior studies had explored the academic motivations, grit, and self-control profiles simultaneously among university students. The relevant previous studies that coincided with this study’s main interest and embraced the person-centered approach in examining students’ motivational attributes include academic motivation profiles alone [6, 11, 12, 15, 20, 48], and grit and self-control together [7]. This study, which aimed to explore the Arabic university students that might be representative to some extent of the views of students in the Middle East, extends the findings of Hong et al. [6] that surveyed American students, Healy et al. [15] that investigated British students, Litalien et al., [11] and Ratelle et al., [48] that examined Canadian undergraduates, Vansteenkiste et al., [12] that studied Belgian students, and the conclusions of Yang et al. [7] and Wang et al., [20] that studied Singaporean undergraduates and high school students, respectively.

In detail, Hong et al. [6] showed four profiles of undergraduates based on four outcomes of academic motivation (i.e., self-efficacy, achievement goals, value, and cost). These profiles were [1] High cost (i.e., was characterized by high cost and low self-efficacy, goals, and values; 9.1%), [2] Moderately motivated (i.e., had students with an average level of all motivation; 37.7%), [3] High goals (i.e., contained students with higher achievement goals compared to other profiles; 41.9%), and [8] Mastery-driven (i.e., had students with low-performance achievement goals and avoidance goals; 11.3%).

Among Canadian undergraduates, Litalien et al. [11] identified five latent profiles, which are [1] Knowledge-oriented (i.e., learners reported moderately high levels of intrinsic motivation to know, low levels of amotivation, and average levels of other types of motivations, 17.6%), [2] Controlled (i.e., students with moderately high levels of have-to motivation, moderately low levels of amotivation, and average levels on the want-to motivations, 26.0%), [3] Multifaceted (i.e., students with moderately high to very high levels on most types of motivation and low levels of amotivation, 15.0%), [8]. Unmotivated (i.e., students who reported low to moderately low scores on most types of motivation, but a moderately high score of amotivation, 25.4%), and [32]. Hedonist (i.e., a very high level of intrinsic motivation to experience stimulation and amotivation, moderately high levels of identified regulation, average levels of intrinsic motivation to know, intrinsic motivation to accomplish, and external regulation, and a very low level of introjected regulation; 16.0%).

Another study examined the latent heterogeneity in grit, self-control, and other attributes influencing addictive smartphone use (i.e., depression, stress, loneliness, fear of missing out, mindfulness, rive, reward responsiveness, and fun-seeking) among college students [7]. The findings revealed three profiles, which are: [1] the gritty, self-controlled, and mindful profile (i.e., learners reported higher means of grit, self-control, and mindfulness, and lower of depression, stress, loneliness, and fear of missing out, reward responsiveness, and fun-seeking), [2] emotionally distressed profile (i.e., had students with higher levels of depression, stress, loneliness, and fear of missing out, and low levels of other protective, inhibitive, and actionable attributes), and [3] the approach sensitive profile (i.e., had students with higher levels of behavior inhibitive system and lower values emotional, protective, and approach attributes).

Furthermore, prior studies also highlight the role of demographic information in predicting the members of estimated profiles. Hong et al. [6] found that three demographic information (i.e., gender, age, underrepresented minority) predicted profile membership. Related to gender, more males were classified in the high-cost and mastery-driven groups compared with the moderately motivated and high-goals groups. Concerning age, Hong et al. [6] found that older students exhibited a significantly higher likelihood of membership in the high goals and mastery-driven profiles compared to other profiles. As well, underrepresented minority students were more likely to be members of high goals relative to other profiles. Litalien et al. [11] found no differences between profiles across gender and age groups.

Previous research also has emphasized the value of examining the role of estimated profiles in predicting other students’ outcomes. Wang et al., [20] four distal outcomes (i.e., effort, value, competence, and time spent on Math beyond homework) were predicted by profile membership. Meaning, students in Profile 3 with higher intrinsic motives spent more time doing Math tasks, showed greater effort, held higher value for math, and showed better competence compared with other profiles. Furthermore, Hong et al. [6] revealed that profile membership predicted academic performance and the use of metacognitive processing. Another study found significant differences between academic motivation latent clusters in learning outcomes (i.e., cognitive processing, and meta-cognitive regulation including test anxiety, time use, and meta-cognitive strategy), determination (i.e., effort regulation and procrastination), and teacher need support (i.e., autonomy support, structure, and involvement; 63).

The current study was mainly interested in understating how the estimated latent profiles differ in ambiguity tolerance. The support for selecting ambiguity tolerance as a distal outcome was lent from the findings of one of the variable-centered research conducted by Varasteh et al. [49]. In a path analysis model, Varasteh et al. [49] revealed significant positive associations among academic motivation as presented by self-regulation, deep learning, and tolerance of ambiguity, which influenced positively academic achievement among undergraduates.

### Study Aim and Research Questions

**Fig. 1:**
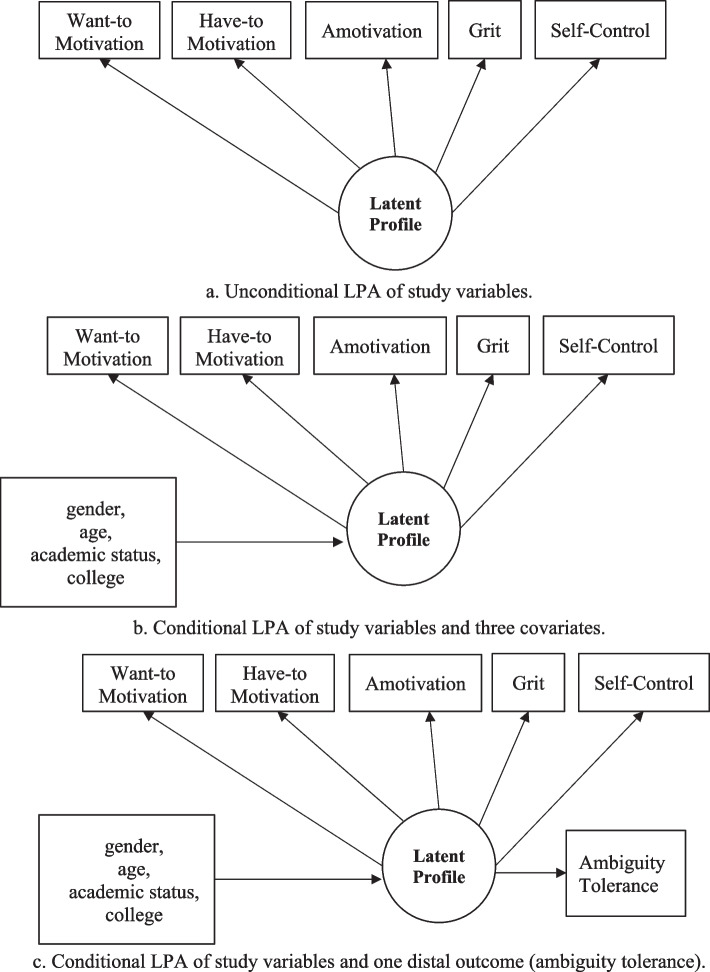
Three Step LPA Approach of Modeling Heterogeneity in the Study Variables. **a** Unconditional LPA of study variables. **b** Conditional LPA of study variables and three covariates. **c** Conditional LPA of study variables and one distal outcome (ambiguity tolerance)

**Fig. 2:**
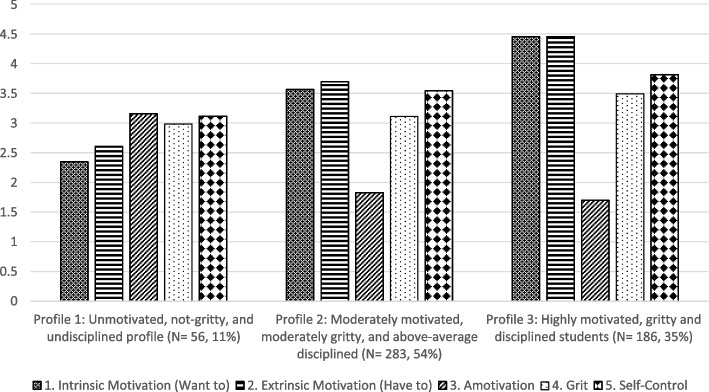
Unconditional three-profile structure of study variables. Note: The X-axis represents the categorical latent profiles. The Y-axis represents the mean response of study variables (i.e., ranging from 1 to 5). The distinctive bars represent three types of academic motivation (i.e., intrinsic motivation, extrinsic motivation, and amotivation), grit, and self-control (i.e., observed indicators)

 The present study, therefore, sought to investigate the latent heterogeneity based on five positive psychology attributes (three academic motivations, grit, and self-control) among university students in the Sultanate of Oman and Egypt, controlling for specific background covariates (i.e., gender, age, academic status, and college) using a three-step LPA approach, which includes fitting: (1) Unconditional LPA (see graph *a* in Fig. 1), (2) Conditional LPA (see graph *b* in Fig. 1), and (3) Conditional LPA with distal outcomes (see graph *c* in Fig. 1).

To this end, the present study sought to answer the following questions:

1. To what extent do different latent profiles of study variables (want-to motivation, have-to motivation, amotivation, grit, and self-control) exist among university students?
2. To what extent are students’ demographic variables (i.e., gender, age, academic status, and college) associated with the estimated latent profiles?
3. Do the estimated latent profiles predict tolerance ambiguity among university students?

## Method

### Participants

After obtaining ethical approval, a complete sample of 525 university students was collected in the Sultanate of Oman and Egypt. There were 133 (25.30%) males and 392 (74.70%) females. The mean age of the sample was 22.64 (*SD* = 4.89). The students varied in their educational degrees, academic years, colleges and majors. The majority of the students hold Bachelor’s degrees (*n* = 411; 78.30%). The minority of students hold higher graduate degrees (*n* = 114; 21.70%). The minority of students studied in scientific college (*n* = 372; 70.90%) and art college (*n* = 142; 27.00%).

### Measures

The online survey had five sections, which include: [1] Demographic information, [2] the Arabic Academic Motivation scale (AMS; 1, 62), [3] The Arabic Grit-S scale [2, 50], [8] Brief Self-Control Scale [38, 51], and [32] Short Multiple Stimulus Types Ambiguity Tolerance MSTAT-II [52]. The first section covers different demographic information, including gender, age, study level, academic year, college, and major.

The second scale, the Arabic AMS scale, was a 28-item scale that has three dimensions (i.e., Want-to, Have-to, and Amotivation). Examples of items covering several reasons for learning are “Because I experience pleasure and satisfaction while learning new things”, “Because with only a high-school degree I would not find a high-paying job later on”, and “I can’t see why I go to college and frankly, I couldn’t care less”. These items were rated on a 7-point Likert scale in the original scale [1]. However, in this study, the response structure was changed from a 7-point to a 5-point Likert scale, which ranges from “Completely Disagree” (Coded 1) to “Completely Agree” (Coded 5). This approach was convenient considering the sample’s workload, and competing pressures [53] and to lessen respondents’ frustration with answering an online survey [54] which increase response quality and rates. The three subscales had a high to good internal consistency reliability (α = 0.90, 0.86, 0.78, respectively) in the current study, which was comparable to prior studies (i.e., 0.86 to 0.83; 62).

The Arabic Grit-S scale was an eight-item scale that was rated a 5-point scale and consists of two dimensions: consistency of interest (*n* = 4 reversed coded items, e.g., I have been obsessed with a certain idea or project for a short time but later lost interest) and perseverance of effort (*n* = 4 positive items; e.g., Setbacks don’t discourage me). The reliability coefficient of the scale was acceptable (Cronbach’s 𝛼 = 0.60) in the current study.

The third scale, the Brief Self-Control Scale [21], consists of 13 items reflecting general capacity for self-discipline, inclination toward deliberate or non-impulsive action, healthy habits, self-regulation in service to building a strong work ethic, and reliability. The scale has nine negative items. The scale had good internal consistency reliability (α = 0.79) in the current study, which was similar to prior studies (i.e., 0.83 and 0.80; 27; 57).

The last scale, MSTAT-II [52], consists of 13 items and assesses several stimuli including ambiguous stimuli in general, complex stimuli, uncertain stimuli, novel stimuli, and insoluble stimuli. Examples of scale items are “I don’t tolerate ambiguous situations very well”, “I prefer familiar situations to new ones.”, and “I avoid situations that are too complicated for me to easily understand.” All items were rated on a 5-point scale. The reliability coefficient of the scale was acceptable (Cronbach’s 𝛼 = 0.69), which was to some extent similar to the reliability coefficient presented by McLain [52].

### Data Analysis

Two prime analyses were used: an examination of descriptive statistics using SPSS 26.0 and LPA using Mplus 8 [55]. Initial data screening procedures were done, including scrutiny of normality, outliers, and missing data [56]. Under the umbrella of finite mixture modeling, LPA is a person-centered framework that identifies clusters of people by examining the associations between persons, and it is used to model continuous observed variables [57–59].

Nylund-Gibson et al. [60] underlined seven methods to examine the effects of covariates and continuous distal outcomes in the mixture models, including LPA. The most accurate methods are ML three-step [61], LTB three-step [62], two-step method [63], and BCH three-step [64]. The current study adopted the automatic BCH three-step method, which includes: [1] Estimating the most precise number of academic motivations, grit, and self-control profiles by evaluating the fit indices of unconditional LPA models with an increasing number of profiles, [2] Modeling the influences of covariates (e.g., age, gender, and colleges) on the profile membership by applying the conditional LPA model with the finest profile-structure, and [3] Predicting the distal outcome (i.e., ambiguity tolerance) by latent profile membership (i.e., adding auxiliary = distal [BCH] in the Mplus code) by applying conditional LPA with distal outcome model. This method was selected because it calculates the classification errors for each person in Step 2. Furthermore, it uses these errors as weights in Step 3, dropping the likelihood of greater variation in the class membership between steps 1 and 3 [60].

Prior to model fitting, the adequacy of the sample size was examined. Kline [65] recommended that the sample size should be 10 to 20 times as many respondents as parameters and at least 200 individuals to have sufficient power. Additionally, a minimum sample size of 100 to 200 to warrant confidence in the model fit indices [10]. The sample size in the present study met all these criteria, implying the adequacy of the sample size.

Several indices were evaluated to identify the optimal number of latent profiles (e.g., [66, 67]). These indices belong to the following categories: [1] Lo-Mendell-Rubin test (LMR), Vuong-Lo- Mendell-Rubin likelihood test (VLMR), and bootstrap likelihood ratio test (BLRT) from the Likelihood Ratio Tests (LRTs), [2] Entropy estimate, and [3] Akaike information criterion (AIC), Bayesian information criterion (BIC), and sample adjusted BIC (SABIC) from the Information Criteria (IC). For the LRTs, a significant *p*-value implies a refusal of the model with fewer profiles and acceptance of the model with higher profiles. Entropy estimates of 0.80 and above imply high classification [68]. Low IC signals a good fit [69]. Further criteria were considered, including [1] Parsimony interpretability of the model as supported by a substantive theory, [2] Optimal profile size (i.e., > 5%), [3] Average posterior probabilities (i.e., the diagonal values in the matrix of average latent class probabilities for most likely latent class membership should be ≥ 0.70; 23; 66).

## Results

### An Examination of Descriptive Statistics

These statistics were examined (see Table 1). The outliers were identified using the z-score method. In this study, these values were lower than the conservative criteria (i.e., the values of z-scores of all data points should be located between ± 2.58 along the normal curve; [70]) except the age, implying no concern about the outliers. The results showed that normality was met for all variables, except age. Pearson correlation coefficients between the study’s variables were assessed (see Table 2). The hypothesized correlations were statistically significant.

**Table 1:** Descriptive statistics for selected variables (*N* = 525)

Variables	Scale	Sample statistics					
		*M*	*SD*	Min	Max	Skewness	Kurtosis
1. Gender	Dichotomous	1.75	0.44	1.00	2.00	-1.14	− 0.71
2. Age	Continuous	22.64	4.90	18.00	60.00	3.43	15.34
4. College	Categorical	1.28	0.45	1.00	2.00	0.96	-1.09
5. Intrinsic Motivation	Continuous	3.76	0.76	1.00	5.00	− 0.61	0.42
6. Extrinsic Motivation	Continuous	3.85	0.73	1.08	5.00	− 0.82	0.72
7. Amotivation	Continuous	1.94	0.94	1.00	5.00	1.15	0.78
8. Grit	Continuous	3.23	0.60	1.38	5.00	0.11	0.59
9. Self-Control	Continuous	3.60	0.62	1.23	5.00	− 0.21	− 0.05
10. Ambiguity Tolerance	Continuous	3.12	0.53	1.23	4.69	0.05	0.49

**Table 2:** Pearson correlation coefficients between the selected variables (*N* = 525)

Variables	1	2	3	4	5	6	7	8	9	10
1. Gender	-	0.01	0.02	0.05	0.19^***^	0.10^*^	− 0.17^***^	− 0.08	0.20^***^	− 0.01
2. Age		-	0.45^***^	0.04	0.13^**^	− 0.03	− 0.18^***^	0.06	− 0.05	
3. Study level			-	0.13^**^	0.12^*^	− 0.01	− 0.14^**^	− 0.01	0.09^*^	− 0.00
4. College				-	0.05	0.02	− 0.06	− 0.04	0.06	0.04
5. Intrinsic Motivation					-	0.74^***^	− 0.38^***^	0.31^***^	0.35^***^	0.03
6. Extrinsic Motivation						-	− 0.32^***^	0.30^***^	0.24^***^	− 0.03^***^
7. Amotivation							-	0.09^*^	− 0.40^***^	− 0.12^*^
8. Grit								-	− 0.05	− 0.12^**^
9. Self-Control									-	0.29^***^
10. Ambiguity Tolerance										-

^*^ *p* < .05, ^**^ *p* < .01, ^***^ *p* < .001

*Unconditional Models Results.* Several unconditional LPA models with an increasing number of profiles were examined (i.e., one-profile to four-profile; see Table 3). The findings suggested that the three-profile model was a candidate to accurately capture the latent heterogeneity in five outcomes (i.e., three types of motivation, grit, and self-control), as indicated by most fit indices. The three-profile model had the lowest AIC, BIC, and SABIC. The entropy value was 0.82, implying high classification accuracy. The LMR-LRT, VLMR-LRT, and BLRT were significant, supporting the goodness-of-fit for the three-profile model relative to the two-profile model. The average posterior probabilities were optimal (i.e., ≥ 0.70), supporting accurate classification. However, the sizes of all profiles were optimal cut-off (i.e., > 5%), suggesting a good classification. In contrast, the four-profile model had nearly similar AIC, BIC, and SABIC values compared to the three-profile model. Entropy (i.e., 0.82), and average posterior probabilities (i.e., > 0.70) were optimal. However, the size of Profile 4 was smaller than the optimal size (i.e., < 5%) and two fit indices (i.e., LMR-LRT and VLMR-LRT) were not significant for the four-profile model. Overall, these results supported the decision to accept the three-profile model and reject the four-profile model.

**Table 3:** Fit Indices of unconditional LPA models with increasing number of latent profiles

	One-profile	Two-profile	Three-profile	Four-profile
Fit statistics				
LL(No. of parameters)	-2862.77(10)	-2651.87(16)	-2542.45(30)	-2501.76(28)
AIC	5745.55	5335.74	**5118.90**	**5059.52**
BIC	5788.18	5403.96	**5246.80**	**5178.89**
SABIC	5756.44	5353.17	**5151.57**	**5090.02**
Entropy	-	0.86	**0.82**	**0.81**
LMR-LRT(*p*)	-	410.87(0.00)	**213.94(0.00)**	79.35(0.24)
VLMR-LRT(*p*)	-	-2862.77(0.00)	**-2638.13(0.00)**	-2542.49(0.23)
BLRT(*p*)	-	-2862.77(0.00)	**-2638.13(0.00)**	-2542.49(0.00)
Profile size (%) P1	525(100%)	91(17%)	**59(11%)**	275(52%)
P2		434(83%)	**282(54%)**	58(11%)
P3			**184(35%)**	168(32%)
P4				25(4%)

*LL *Lilelihood, *AIC *Akaike Information Criterion, *BIC *Bayesian Information Criterion, *SABIC *Sample Adjusted BIC, *LMR-LRT *Lo-Mendell-Rubin test, *VLMR-LRT *Vuong- Lo-Mendell-Rubin Likelihood Test, *BLRT *Bootstrap Likelihood Ratio Test

Statistical significance alone is not adequate when determining the best number of profiles. Lending support from the substantive theory is a necessity. The three-profile structure obtained in the current study was supported by Ratelle et al., [48] and Healy et al., [15] that examined similar academic motivations (i.e., want-to, have-to, and a motivation). The interpretability of the four-profile structure was debatable. Three prior studies substantiated the presence of four profiles [6, 12, 20]. However, Hong et al. [6] examined a different set of variables (i.e., self-efficacy, achievement orientation goals, values, and costs). Wang et al., [20] identified profiles among high school students, which differ from the current study sample (i.e., university students). Vansteenkiste et al. [12] adopted cluster analysis, which varies from this study’s analytic framework (i.e., LPA), which cannot be informative about the exact number of latent profiles.

Considering the above-mentioned empirical evidence and regarding the first research question, the current study concluded that the three-profile structure had the best model fit and the most accurate profile enumeration. The retained three-profile model is presented in Fig. 2. Table 4 shows the average values of five outcomes for each profile. Profile 1 included 11% of students (*n* = 59) with high amotivation, low want-to and have-to motivation, and low grit and self-control. This profile was the most problematic and was named as **“** *Unmotivated, not gritty, and undisciplined*”. Students in Profile 2 had medium grit and moderate to high self-control and were moderately motivated, as characterized by above-average have-to motives, average want-to motives, and low amotivation, representing 54% of the sample (*n* = 283). This profile was named as “*Moderately motivated, moderately gritty, and above-average disciplined*”. Lastly, 35% of students (*n* = 186) reported the highest means on four observed indicators (i.e., want-to, have-to, grit, and self-control) and the lowest amotivation in Profile 3. This profile was prime and was named as “*Highly motivated, gritty and disciplined students*”.

**Table 4:** The unstandardized parameters of the unconditional three-profile model

Study variables	Profile 1: Unmotivated, not gritty, and undisciplined		Profile 2: Moderately motivated, disciplined with medium grit		Profile 3: Highly motivated, gritty and disciplined students	
	M	SD	M	SD	M	SD
1. Intrinsic Motivation (Want to)	2.36^***^	0.11	3.57^***^	0.04	4.46^***^	0.05
2. Extrinsic Motivation (Have to)	2.61^***^	0.11	3.70^***^	0.05	4.46^***^	0.04
3. Amotivation	3.16^***^	0.19	1.83^***^	0.06	1.70^***^	0.07
4. Grit	2.98^***^	0.09	3.11^***^	0.04	3.49^***^	0.05
5. Self-Control	3.12^***^	0.08	3.55^***^	0.04	3.83^***^	0.05

^*^ *p* < .05, ^**^ *p* < .01, ^***^ *p* < .001

#### Conditional Latent Profile Analysis Results

In addressing the second research question, a conditional three-profile model was used to investigate the associations between four covariates (i.e., gender, age, study level, and college) and latent profile membership (see Table 5). The multinomial logistic regression coefficients identified the likelihood of belonging to a particular latent profile relative to a reference profile, which was in this case Profile 3 (i.e., Highly motivated, gritty, and disciplined students; see Table 5). The were two reasons for selecting Profile 3 as the reference profile. First, Profile 3, which has highly motivated students, provided the best interpretation of the results, aligning with prior studies [57]. Second, it clearly articulated the characteristics of the profiles that need more focus and care (i.e., Profile 1 with low motives and Profile 2 with intermediate motives). Odds were estimated for the significant coefficients, facilitating the interpretation of the findings [68]. Gender, study level, and college were negatively associated with classification in Profile 1 (i.e., Unmotivated, not-gritty, and undisciplined). The odds of classification in Profile 1 increased by 0.31, 0.16, and 0.99 when the students were males, had bachelor’s degrees, and studied in scientific colleges, respectively relative to the students in the reference group. In contrast, none of the covariates were significant in predicting Profile 2 membership relative to the reference group.

**Table 5:** Regression estimates and multinomial logistic odds of theory-selected covariates on the two latent profiles relative to a reference profile (i.e., Profile 3: Highly motivated, gritty, and disciplined students)

Fit statistics	Profile 1 (Unmotivated with low grit and self-control)		Profile 2 (Moderately motivated, disciplined with medium grit)	
	Regression coefficients	Odds	Regression coefficients	Odds
Gender	-1.17^*^	0.31^***^	− 0.38	
Age	0.00		0.00	
Study level	-1.08^*^	0.16^***^	− 0.33	
College	− 0.01^*^	0.99^*^	− 0.00	

^*^ *p* < .05, ^**^ *p* < .01, ^***^ *p* < .001

The third research question addressed the prediction of ambiguity tolerance, as a continuous distal outcome, by latent profile structure using an automatic BCH three-step approach. The findings were summarized in Table 6. The findings showed a significant difference between the profiles in ambiguity tolerance. Particularly, students in Profile 3 (*Highly motivated, gritty, and disciplined students*) had significantly higher ambiguity tolerance compared to students in Profile 2 (Moderately motivated, disciplined with medium grit). Unexpectedly, no significant difference was found between Profile 1 (Unmotivated with low grit and self-control) and Profile 3 (*Highly motivated, gritty, and disciplined students*).

**Table 6:** Differences between profile-specific means of ten distal outcomes by automatic BCH three-step approach

Fit statistics	*Approximate χ* ^*2*^ values for mean comparisons	Profile (Distal mean)	Means differences	
			Profile 1	Profile 2
Ambiguity Tolerance	Overall test = *χ* ^*2*^ [2] = 6.35, *p* < .05			
	Profile 1 vs. 2 = *χ* ^*2*^ [2] = 0.44, *p* = .51	P1low (3.08)	0	
	Profile 1 vs. 3 = *χ* ^*2*^ [2] = 1.06, *p* = .30	P2in (3.03)	0.05	
	Profile 2 vs. 3 = *χ* ^*2*^ [2] = 6.35, *p* < .05	P3hi (3.17)	− 0.09	− 0.14

## Discussion

Even though the quantity and quality of academic motivations, grit, and self-control shaped the quality of students’ academic and personal outcomes, well-documented divergence in the students in adopting productive academic motivation and practicing the optimal level of grit and self-control [3; 20, 52, 70], resulting in long-lasting effects students’ learning, momentary and future paths, as indicated by numerous variable-centered studies [2, 9, 59]. Furthermore, several studies acknowledge considerable heterogeneity in students’ academic motivation [6, 11, 15, 20], grit, and self-control [7]. Treating students as a homogenous group in terms of three academic motivations, grit, and self-control, as is the case in variable-centered studies, may result in biased and inaccurate estimation of the associations between these variables and students’ academic outcomes, including ambiguity tolerance in case of complicated and vague learning experiences. This entails the value of adopting person-centered approaches to detect students’ profiles depending on the level of five variables (want-to motives, have-to motive, amotivation, grit, and self-control) simultaneously. Therefore, this study had three purposes: [1] Model the latent variability in five variables and accurately enumerate the profile structure using unconditional LPA, [2] Examine the relationships among profile membership and four demographic information (i.e., gender, age, academic status, and college) using conditional LPA, and [3] Investigate the difference between obtained profiles in ambiguity tolerance using automatic BCH three-step LPA.

The findings of unconditional LPA revealed substantial latent heterogeneity in study variables, indicating that students cannot be deemed as a homogenous group in terms of their academic motives, grit, and self-control. A comparison of several models with an increasing number of profiles (i.e., two, three, and four profiles) showed that the four-profile and three-profile were candidates for capturing the latent heterogeneity, as indicated by the similar and optimal model fit indices. However, the four-solution was rejected due to the small profile size (i.e., < 5% for Profile 4) and lack of substantive theoretical support [15, 48], hinting at poor categorization and questionable interpretability.

The results concluded that the three-profile solution was the best-fit model with accurate profile enumeration. The three profiles were named, as follows: [1] *Unmotivated, not-gritty, and undisciplined profile* (i.e., students with high amotivation, low want-to and have-to motivation, low grit, and self-control), [2] *Moderately motivated, moderately gritty, and above-average disciplined* Profile (i.e., students with a medium grit and moderate to high self-control and were moderately motivated, as characterized by above-average have-to motives, average want-to motives, and low amotivation), and [3] *Highly motivated, gritty and disciplined students* Profile (i.e., students who reported the highest means on four observed indicators [i.e., want-to, have-to, grit, and self-control] and lowest amotivation). This novel finding fills the gap in the literature by providing tangible evidence of latent heterogeneity in academic motivation, grit, and self-control. As well, the current study extended this line of research that adopted the person-centered approach when analyzing students’ motives [6, 11, 15, 48] or grit and self-control [7]. The three-profile structure that arose from the current study, while believed to be valid, differs from the five-profile that were detected by Litalien et al. [11] and the four-profile of academic motives that were found by other three prior studies [6, 12, 20]. The discrepancy in the number of profiles can be attributed to the fact that the prior study examined different outcomes than the one that was investigated in the current study (e.g., self-efficacy, achievement orientation goals, values, and costs; 33), distinct samples (e.g., high school students; 66), and divergent analytical framework (e.g., cluster analysis; 63).

The conditional three-profile LPA revealed that theory-driven covariates predicted profile membership. The strength of these association coefficients fluctuates across the three latent profiles. Gender, study level, and college were negatively associated with classification in Profile 1 (i.e., Unmotivated, not gritty, and undisciplined). The odds of students being in Profile 1 as opposed to the comparison profile, Profile 2 (i.e., Highly motivated, gritty, and disciplined), were higher by 0.31, 0.16, and 0.99 when the students were males, bachelors, and studied in scientific colleges, respectively. Concerning gender, this result is consistent with Hong et al. [6], which showed that more males were classified in the high-cost and mastery-driven groups compared with the moderately motivated and high-goals groups, implying that males were more likely to report lower performance and high perception of costs. Yet, it contradicted Litalien et al. [11] that proved the similarity of the estimated profiles in the male and female samples. Furthermore, this study provides novel findings about the role of study level and college in identifying the distinctive features of the profiles extending the findings of prior studies (e.g., 31, 33, 41, 51, 71). These findings emphasize the necessity for designing interventions to strengthen students’ motives, adopting the proper reasons for learning, and empower the ability to practice self-control and grit among male students, bachelors and studied in scientific colleges, which echoes the findings of prior studies [6, 7]. In contrast, none of the covariates were significant in predicting Profile 2 membership (i.e., Moderately motivated, moderately gritty, and above-average disciplined) relative to the reference group (i.e., Highly motivated, gritty, and disciplined), suggesting a similarity in students characteristics across two profiles.

An additional novel finding from the current study is that profile membership predicted students’ ambiguity tolerance by demonstrating significant differences between the two profiles. That is, students in Profile 3 (*Highly motivated, gritty, and disciplined students*) had significantly higher ambiguity tolerance compared to students in Profile 2 (i.e., Moderately motivated, moderately gritty, and above-average disciplined) agreeing with several prior studies [21, 23, 44]. No significant differences were found between Profile 1 and Profile 2. Surprisingly, no significant difference was found between Profile 1 (*Unmotivated with low grit and self-control*) and Profile 3 (*Highly motivated, gritty, and disciplined students*) in ambiguity tolerance. This nonsignificant difference can be attributed to the fact that demotivated students tend to adopt maladaptive strategies including defensive pessimism and self-handicapping [71]. Examples of these unproductive strategies include avoidance, denial, deliberately withholding effort, low task value, less care about learning experience, procrastination, lack of practice, and reporting illness [72]. Such low care about learning experience might lead to indifference state about the degree of ambiguity in learning experience.

### Implications and Limitations

The current study had several theoretical implications. This study’s results shed light on the considerable heterogeneity in academic motives, grit, and self-control among Middle Eastern university students. The study also revealed that various theory-selected covariates (gender, study level, and colleges) detected the characteristics of students within each profile. A clear difference in ambiguity tolerance was found based on the level of academic motives, grit, and self-control. Furthermore, the present study delivered many valuable pragmatic applications, suggesting solutions to the ramifications of improper motives, low grit, and low self-control (e.g., [4, 39]). Educational interventions that encourage adopting adequate motives and empower grit and discipline in Profile 1 (i.e., *Unmotivated, not gritty, and undisciplined*) are suggested. Initiatives are recommended mainly for males, bachelors, and studied in scientific colleges.

This study also has numerous limitations. It studied the views of only Omani and Egyptian students, which can limit the generalizability of the findings to the Middle East only. The current study investigated only four academic student-related covariates (i.e., gender, age, academic status, and college). Examining other cognitive student-related and contextual variables (e.g., Family-related variables) would afford in-depth insights into the factors inducing the heterogeneity in academic motives, grit, and self-control. The present study analyzed cross-sectional data, which provides only a snapshot of the level of studied variables at a specific point. This restricted framework does not essentially scrutinize the changes in studied variables across time. Thus, conducting a longitudinal study is highly preferred [73].

Endorsed topics for future research include [1] Conducting quasi-experimental studies that study the effectiveness of educational interventions to facilitate adopting the optimal motives and practicing self-control among males, bachelors and studied in scientific colleges, [2] examination of the effects of contextual family-related, school-related, and cognitive-related covariates on the heterogeneity of studied variables among university students; and [3] The implementation of a longitudinal study that explores the fluctuations in the studied variables across time.

## Conclusion

The students whose data were analyzed in the current study showed significant heterogeneity in their academic motives, grit, and self-control, forming three latent profiles. The features of students in these profiles were significantly predicted by several demographic variables. Males, with bachelors and studied in scientific colleges were more likely to be classified in the unmotivated profile with low grit and self-control. Simultaneously, a significant difference in ambiguity tolerance was found between the highly motivated and moderately motivated profiles. Implementing appropriate and effective Initiatives was recommended to enhance student academic outcomes.

## Data Availability

The datasets generated during and/or analyzed during the current study are not publicly available to maintain the privacy of the respondents but are available from the corresponding author on a reasonable request.
